# Stimulus Dependency of Object-Evoked Responses in Human Visual Cortex: An Inverse Problem for Category Specificity

**DOI:** 10.1371/journal.pone.0030727

**Published:** 2012-02-17

**Authors:** Britta Graewe, Peter De Weerd, Reza Farivar, Miguel Castelo-Branco

**Affiliations:** 1 Department of Cognitive Neuroscience, Faculty of Psychology & Neuroscience, Maastricht University, Maastricht, The Netherlands; 2 Visual Neuroscience Lab, Institute of Biomeedical Research in Light and Image, University of Coimbra, Azinhaga de Santa Comba, Coimbra, Portugal; 3 Donders Institute for Brain, Behaviour and Cognition, Radboud University, Nijmegen, The Netherlands; 4 Harvard Medical School and Massachusetts General Hospital, A. Athinoula Martinos Center for Biomedical Imaging, Charlestown, Massachusetts, United States of America; 5 Visual Neuroscience Lab, IBILI, University of Coimbra, Azinhaga de Santa Comba, Coimbra, Portugal; National Institute of Mental Health, United States of America

## Abstract

Many studies have linked the processing of different object categories to specific event-related potentials (ERPs) such as the face-specific N170. Despite reports showing that object-related ERPs are influenced by visual stimulus features, there is consensus that these components primarily reflect categorical aspects of the stimuli. Here, we re-investigated this idea by systematically measuring the effects of visual feature manipulations on ERP responses elicited by both structure-from-motion (SFM)-defined and luminance-defined object stimuli. SFM objects elicited a novel component at 200–250 ms (N250) over parietal and posterior temporal sites. We found, however, that the N250 amplitude was unaffected by restructuring SFM stimuli into meaningless objects based on identical visual cues. This suggests that this N250 peak was not uniquely linked to categorical aspects of the objects, but is strongly determined by visual stimulus features. We provide strong support for this hypothesis by parametrically manipulating the depth range of both SFM- and luminance-defined object stimuli and showing that the N250 evoked by SFM stimuli as well as the well-known N170 to static faces were sensitive to this manipulation. Importantly, this effect could not be attributed to compromised object categorization in low depth stimuli, confirming a strong impact of visual stimulus features on object-related ERP signals. As ERP components linked with visual categorical object perception are likely determined by multiple stimulus features, this creates an interesting inverse problem when deriving specific perceptual processes from variations in ERP components.

## Introduction

There is a strong ongoing debate on the category specificity of event-related potentials (ERPs). Especially in the case of face stimuli, many studies have interpreted their results in favour of category-specific processing. While differences in amplitude related to object categories have been reported as early as 100 ms (P100) [1] and in later components such as the N250 [2], the ‘N170’, a negative potential over occipital-temporal sites is the most famous and well-studied peak showing a larger amplitude for faces than for other object categories [3]–[6]. Similarly, the discovery of the face-inversion effect demonstrating a delayed and enhanced N170 to inverted faces but not to inverted objects [7], [8] has contributed to a consensus on the face specificity of this peak. Stimuli in prior studies of category-specific ERP components typically consisted of photographs (in black and white, grey-levels or color) or schematized images (line drawings, cartoons or Mooney stimuli) (for a review see [9]). However, even if the dimensions that are represented in high-level cortex cannot be reduced to physical features [10], [11], low-level physical properties unavoidably differ between categories and may therefore contribute to differential ERP responses [12]–[15]. Indeed, in the case of the N170, many results characterize a sensitivity to a variety of stimulus manipulations including *feature isolation* [3], *contrast polarity reversal* [16], *spatial frequency filtering* [17], [18] and *stimulus orientation* [19]. While some studies have equated images on global factors such as contrast, luminance level and spatial frequency [14], [20], different object categories have often been compared without using control images that were equated on visual features [4], [5], [21] or by using (scrambled) control images that not only abolished categorical object information but also cue configurations and visual structure [3], [22], [23]. In all of these studies, visual feature properties may have added significantly to the results.

To test the relative contributions of categorical and visual feature variations on ERP responses, we performed two types of experiments. First, we aimed to separate the impact of visual feature and category-related factors on the ERP signal by comparing ERP peaks elicited by structure-from-motion-(SFM) objects and restructured control stimuli containing the same visual feature information (luminance, contrast, SF, motion) (Exp. 1A&B). In SFM stimuli the object percept arises solely from a moving dot pattern with the object being invisible when the dots are stationary. The use of dynamic localized cues [24] avoids contour and other artifacts that would have appeared in restructured, static luminance-defined control images hence making SFM stimuli well-suited for the purpose of the present study. Second, to investigate the impact of visual feature variations on object-related ERPs parametrically, we manipulated the stimulus depth range in SFM-defined and luminance-defined faces and chairs by ‘flattening’ these objects (Exp. 2B&C). The use of objects defined by both motion and luminance cues enabled us to strengthen the generality of our findings.

## Materials and Methods

### Participants

All participants (*N* = 48, ages 22–31, 22 males and 26 females) had normal or corrected-to normal vision and no history of neurological disorders. The experiment was approved by the local Ethics Committee of the University of Coimbra and before the start of the experiment written and oral informed consent was obtained from all participants in accordance with the Helsinki Declaration. A total of 27 participants took part in EEG recordings, distributed over 4 experiments (Exp. 1A, 1B, 2B, 2C). The remaining 21 participants performed a psychophysical control experiment (Exp. 2A).

### Procedure

Participants were seated in a dimly lit sound-attenuated cabin viewing stimuli subtending ∼13° of visual angle horizontally and ∼10° vertically. Viewing distance was 80 cm and stimuli were always presented at the center of the screen of a CRT monitor (resolution 1024*768, refresh rate 60 Hz). Stimuli were delivered using Presentation 12.1 (Neurobehavioral systems).

### Experiment 1: Categorical stimuli versus matched control stimuli

The ERP responses evoked by SFM categorical and control stimuli were investigated in 14 participants, in two separate but comparable experiments (Experiment 1A: 8 participants, 5 female, 3 male; Experiment 1B: 6 participants, 3 female, 3 male).

### Experiment 1A

#### Stimuli

**Figure 1:**
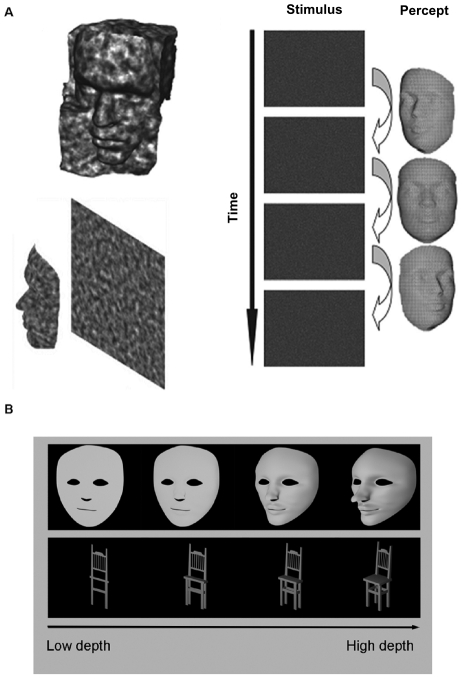
Stimuli used in the present study. **A** Illustration of a structure-from-motion object stimulus. The percept of a rotating face emerges from a moving dot pattern in the absence of other visual cues (used in Exp. 1A, 1B, 2A, 2B) **B** Static luminance-defined faces and chairs with varying depth ranges (used in Exp. 2C). 3D structure was manipulated by computing views of ‘flattened’ objects causing variations in complexity and strength of a number of visual features (see Materials and Methods). (Figure adapted from [24]).

SFM stimuli were videos of SFM-defined faces, chairs, and control stimuli (for details see [24]). Face stimuli consisted of 10 laser-scanned facial surfaces taken from the Max-Planck Face Database, rendered with a volumetric texture map ensuring uniform texture density across the surface (as described in detail in [25]). Shadows and shading were removed and the faces were rendered against a similarly textured random-dot background. During stimulus presentation, the face rotated from −22.5 degrees to 22.5 degrees centered at the frontal plane in one cycle (Figure 1A) with the rotation being captured in a video that lasted 860 ms (26 frames). The 10 chair stimuli were obtained from a chair model database and were rendered in exactly the same manner as the faces. Control versions of the two stimuli were constructed by cutting the rendered whole object (face or chair) videos in the horizontal plane into ten blocks and restructuring their positions within the object boundaries. Importantly, this manipulation did not introduce localized cues (high spatial frequency (HSF) noise confounds) but resulted in control stimuli that share many of the visual features of the original videos (including luminance, contrast, texture, spatial frequency, motion, and strength of depth and curvature cues in contours and surfaces). Thus, intact and control images were equated in visual features, with the intact stimuli showing a recognizable object, and the restructured control images a meaningless object entity.

#### Task

It has previously been shown that for tasks in which participants attend to face identity, the N170 amplitude is enhanced for familiar compared to unfamiliar faces [26], [27]. Participants were therefore trained to associate names with 10 SFM faces and 10 SFM chairs prior to the start of the EEG experiment. Training was halted when at least 80% of the responses were correct. To ensure sustained attention and task engagement during the EEG recordings, participants were asked to maintain eye fixation and were presented with a stimulus sequence consisting of an SFM stimulus (860 ms), a fixation period (1 sec) and the presentation of a name from the respective category (face or chair name; 1 sec). In the 1 sec response period following each trial, they then indicated by a button press whether according to the face-name/chair-name encoding they had done in the previous training, the presented name corresponded to the SFM stimulus (left button: yes, right button: no). Participants were asked to respond as accurately as possible and did not receive feedback on their responses. For consistency, button presses were also given in the control SFM stimulus conditions for which faces or chairs were however unidentifiable, hence resulting in random responses. The experiment was divided into 5 self-initiated runs of 12 minutes each. There were 250 trials for each stimulus category resulting in a total of 1000 trials which were presented in random order.

### Experiment 1B

#### Stimuli

Here, the SFM stimuli used in experiment 1A (face, chair, and associated control stimuli) were shortened to 160 ms (5 frames) and rotated from −4.3 degrees to 4.3 degrees. Prior to the EEG recordings we created stimuli of 2 different durations (100 ms and 160 ms) and tested the effect of duration on psychophysical task performance.

#### Task

Participants were asked to perform a simple categorization task in which they indicated by 3 alternative button presses whether the SFM stimulus contained a face, chair or a meaningless object. Thus, in this task object-related processes that might have infiltrated ERP responses to restructured control stimuli (e.g., imagery) are minimized, both because of the unspecific categorization task that no longer required identification of individual exemplars, and because of the explicit categorization of the restructured control stimuli as ‘meaningless’. The two changes compared to experiment 1A (shortened duration and categorization task) aimed to reveal a larger contribution of bottom-up processing and to minimize effects of high-level cognitive factors. The response period was increased to 1.5s, relaxing the time pressure for responding.

### Experiment 2: Parametric variation of visual features in categorical stimuli

In experiment 2A, there were 21 participants (11 female, 10 male); in experiment 2B, 8 participants (5 female, 3 male); and in experiment 2C, 5 participants (2 female, 3 male).

### Experiment 2A

#### Stimuli

Depth range was varied for a single SFM face and a single SFM chair stimulus (duration 160 ms, see exp. 1 for details on the stimuli). This global manipulation of the elementary variable ‘depth’ resulted in new stimuli (parameterized in terms of anterior-posterior range) that were randomly presented at 3 levels of depth (10%, 30%, 90%, expressed as a percentage of the un-manipulated depth range). The change in depth caused variations in complexity and strength of a number of visual features, as can be seen in analogous depth-related variations in luminance-defined stimuli in Figure 1B. For simplicity, this manipulation will from hereon be referred to as ‘depth range manipulation’.

#### Task

Participants performed a psychophysical task (without EEG measurements) in which categorization performance was tested under varying depth levels of the SFM stimuli. Participants were instructed to distinguish between SFM face and chair stimuli by pressing one of two buttons in a 1.5 sec response period. There were 10 trials per condition.

### Experiment 2B

#### Stimuli

As in experiment 2A, depth range was varied for a single SFM face and a single SFM chair stimulus (duration 160 ms, see exp. 1 for details on the stimuli). Here, we used 4 different depth levels (10%, 30%, 60% and 90% of the un-manipulated depth range).

#### Task during EEG recording

Similar to the psychophysical control task (Exp. 2A), single SFM faces or single SFM chairs were displayed in random order at the 4 depth levels. There were 120 trials per condition with participants indicating by button press in a 1.5 sec response period which stimulus category they had perceived (face or chair).

### Experiment 2C

#### Stimuli

Luminance-defined static face and chair stimuli (Figure 1B) were constructed using Blender software. The resulting monochromatic meshes were depth-manipulated along the y axis which in the face meshes implied variations in the convexity of facial features, while in the chair meshes it caused variations in the length of the seat and in the width of the legs and backrest. Thus, as was the case for SFM stimuli, this parametric manipulation varied the richness, strength and complexity of the stimulus/object cues. In the absence of changes in a participant's ability to categorize the stimuli, changes in ERP responses are likely to be driven by variations in one or more of such visual features, which would be informative for the question whether responses to object stimuli are driven by visual feature information or by categorical aspects. By including the luminance-defined stimuli, we aimed to answer this question for both SFM and luminance-defined object stimuli. As in experiment 2B, we used 4 different depth levels (10%, 30%, 60% and 90%). Stimuli were displayed for 500 ms (∼15 frames).

#### Task during EEG recording

Participants indicated by button presses in a 1 sec response period which category they had perceived (face or chair). There were 120 trials per condition.

#### ERP Recording and Analysis

Continuous EEG data for experiment 1 were recorded with a NeuroScan SynAmp system at a sampling rate of 2000 Hz from 64 scalp electrodes embedded in a NeuroScan Quik-Cap. Two EKG channels monitored heart rate and four bipolar facial electrodes, positioned on the outer canthi of each eye and in the inferior and superior areas of each orbit, monitored horizontal and vertical EOG (HEOG and VEOG), respectively. The impedance of each electrode was adjusted to less than 10 kΩ. The signal was amplified using Synamps Amplifiers and recordings were made using CPz as an online reference. Following a system upgrade, experiments 1B and 2B, C were conducted with a 128-channel Neuroscan system under equal conditions and settings.

#### Data Analysis

After recording, data were analyzed offline using the Scan 4.3 Edit Software. The continuous data files were downsampled to 500 Hz and digitally low- and highpass filtered (30 Hz, 12 dB/oct and 1 Hz, 12 dB/oct, respectively). Artifact rejection was performed automatically, removing epochs with amplitudes exceeding +/−75 µV and by subsequent visual inspection. Eye movements were corrected for by the blink-noise-reduction option. Artifact free continuous data were then segmented into epochs ranging from −200 ms to 500 ms after stimulus onset for all conditions. The epochs were baseline corrected based on the time interval −200 ms before stimulus onset and subsequently averaged per experimental condition.

#### Peak Analysis

For each participant, 3 peaks were automatically detected and included in the analysis. P100 was determined as the most positive peak between 70 and 140 ms and N2 as the most negative peak between 140 and 200 ms (140–180 ms in the parietal cluster). At later latencies, analysis revealed a large negative peak at ∼250 ms (defined as most negative peak between 210–280 ms; 80–280 ms in the parietal cluster). For the static stimuli in experiment 2C, the N170 peak was defined as the most negative peak between 140 and 210 ms. Based on previous studies and our focus on visual object recognition, statistical analysis was restricted to occipital, posterior temporal and parietal regions. Since the P100 is not the focus of our interest here and analysis in the occipital cluster did not reveal any relevant results, the figures only display ERP results from the parietal and posterior temporal cluster.

For the amplitude and latency analysis, clusters of electrodes were defined, yielding a regional weighted average of the electrodes showing the largest amplitudes and hence optimally revealing the peaks of interest. The occipital cluster included electrodes O1 and O2 (in the 128-channel system 20,21,42,43,44,45 on the left, corresponding sites on the right), the posterior temporal cluster TP7, P7, P7, P05, PO7 over the left hemisphere and corresponding sites on the right (in the 128-channel system 17,18,19,20,21,22,23 on the left and corresponding sites on the right) and the parietal cluster P3 and P4 (in the 128-channel system electrodes 39,40,47,48 on the left and corresponding sites on the right). For the statistical analysis, a repeated measures GLM was applied on the amplitudes and latencies of all components of interest (based on our research question, in exp. 2 only peak amplitude values were included in the analysis). Where applicable, p-values were corrected for non-sphericity using the Greenhouse-Geisser or Huynh-Feldt correction (for simplicity, the uncorrected degrees of freedom are presented) and p-values for multiple comparisons were Bonferroni-corrected.

#### Source localization

Brain generators were estimated over the time period from −100 to 500 ms using a distributed, linear solution to the inverse problem, based on the sLORETA method (standardized low resolution brain electromagnetic tomography, [28]). This method has been shown to have no localization bias in the presence of measurement and biological noise [29] and takes several neurophysiologic and anatomical constraints into account. Current source density maps were constructed assuming multiple simultaneously active sources of a priori unknown location and making no assumption regarding the number or location of active sources. For the source estimation, individual MR data were used to create a Boundary element (BEM) model. Functional ERP data and anatomical MRI scans of individual participants were co-registered using landmarks and applying standard xyz coordinates of the electrode positions on the head. After pre-processing the EEG data, an independent component analysis (ICA) was applied and only the main components (signal to noise ratio SNR>1) were used for the source reconstruction. LORETA solutions were then computed in two fixed time segments (T1: 140–200 ms, T2: 220–290 ms) individually for 5 of the participants who participated in experiment 1B. After establishing the position of each source in Talairach coordinates, anatomical label were obtained with the help of the Talairach Daemon client [30].

## Results

### Experiment 1A: ERP components elicited by SFM categorical stimuli and matched control stimuli

**Figure 2:**
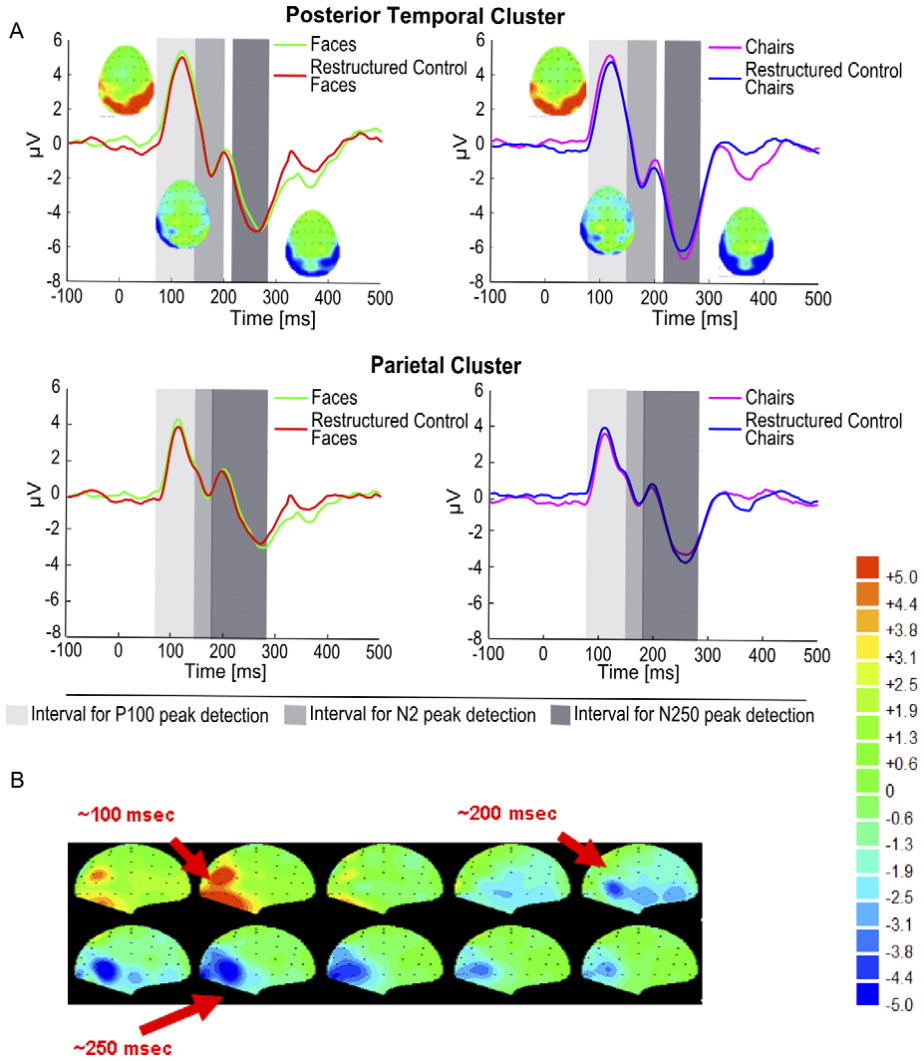
SFM-evoked ERP signals (Exp. 1A). **A** The P1-N2-N250 pattern evoked by SFM stimuli of 860 ms duration is displayed for the posterior temporal and the parietal cluster. Voltage maps at corresponding time points are displayed for SFM face and chair elicited ERP signals. Group averaged ERPs showed no differences between SFM objects and associated restructured control stimuli at the N250 peak. The grey shaded areas indicate the time windows used for peak detection. **B** Voltage maps of the time points of interest for SFM faces. Activity in the occipital cortex is followed by more temporal (negative) activation at later time points (200–300 ms).

Experiment 1 was designed to identify category-related ERP responses elicited by meaningful SFM stimuli of 860 ms duration while participants performed an object identification task (see Materials and Methods). Categories tested were SFM faces and chairs as well as their restructured counterparts (2 categories×2 controls). ERP results revealed a P100-N2-N250 ERP complex in all 4 conditions (Figure 2A). In the statistical analysis, separate repeated measures ANOVAs were computed for both amplitude and latency of each of these components. Factors included in the model were object category (face, chair), stimulus type (intact, restructured) and regional cluster (occipital, parietal, posterior temporal).

For the P100 amplitude, there were no significant main effects of the factors category and stimulus type (*p*>.05) but a significant main effect of cluster (*F*_(2,14)_ = 7.319, *p* = .007), showing that the peaks at the occipital and the parietal clusters were larger than at the posterior temporal cluster. Similarly, the P100 latency analysis revealed no significant main effects of category and stimulus type (*p*>.05) and also no significant main effect of cluster (*p*>.05). ANOVA on the N2 amplitude and latency revealed no significant main effects or interactions (*p*>.05) for any of the factors. For the N250, analysis of peak amplitudes showed no significant main effects (category (*F*_(1,7)_ = 1.553, *p* = .248; scrambling (*F*_(1,7)_ = 2.040, *p* = .191; cluster (*F*_(2,14)_ = 2.902, *p* = .084) and no significant interactions (*p*>.05). In contrast, analysis of the N250 latency revealed a significant main effect of object category (*F*_(1,7)_ = 7.460, *p* = .026) with face-related stimuli eliciting a later N250 than chair-related stimuli. No other main effects or interaction for the N250 latency were significant (*p*>.05). Voltage maps over time in all conditions showed that early occipital activation at around 100 ms was followed by later (posterior) temporal activation (200–300) (Figure 2B).

In summary, the results show that restructuring the SFM stimuli into meaningless objects, i.e. disrupting the object identity information while leaving visual features intact, did not result in a significant modulation of the ERP amplitude, neither at the N2, nor at the N250. In addition, although there was a significantly longer N250 peak latency for SFM face-related stimuli compared to SFM chair-related stimuli, this increase did not differ between meaningful categorical stimuli and meaningless control stimuli. Thus, neither the amplitude nor the latency of ERP components in this experiment provided information specific for meaningful object categories.

### Experiment 1B: ERP components elicited by SFM categorical stimuli and matched control stimuli at brief stimulus durations

Experiment 1A revealed that the difference between meaningful categorical stimuli and meaningless control stimuli matched on visual features was not reflected in the measured ERP components. For the N2, and especially the N250, it is possible that the lack of categorical object-specificity was due to the long stimulus duration and the object identification task, which may have permitted cognitive factors such as imagery to influence the ERPs elicited by the restructured stimuli. Imagery has been found to have a content-specific effect on the ERP within the first 200 ms of stimulus processing [31]. Furthermore, in the case of face processing it has been suggested that the early perceptual stage (e.g. the N170) may be penetrable by top-down effects due to the activation of face representations within the face recognition system (e.g. by showing face ‘primes’) [32], [33]. This raises the question whether the face-name association task in experiment 1 of the present study may have influenced the ERP results. Consistent with this idea is also that top-down effects that may be associated with task performance (leading to enhancement of neural activity) can be seen about 150 ms after stimulus onset in static stimuli [34]. Related effects of attention with a similar timing have been observed in neurophysiological recordings in monkeys [35], [36]. Hence, we shortened the SFM stimuli duration to 160 ms and applied a simple categorization task that limited the possibility for meaningful object-related cognitive processes to affect the processing of the meaningless restructured control stimuli (for details see Materials and Methods).

Psychophysical test data showed that participants needed SFM stimulus exposures of at least 100 ms to identify object categories emerging from the moving dot pattern (<10% error rates at duration 100 ms, results not shown) indicating that the 160 ms durations used in the EEG measurements of experiment 1B were sufficiently long to ensure object categorization. The ERP results indicated that despite the shorter stimulus duration, the pattern P100-N2-N250 as reported in experiment 1A was replicated in experiment 1B. Analysis at the P100 amplitude and latency revealed no significant main effects of object category, stimulus type, or cluster (*p*>.05). For the N2 amplitude, analysis showed a significant main effect of regional cluster (*F*_(2,10)_ = 4.271, *p* = .046) with post-hoc pairwise comparisons revealing that the N2 peak was larger in the posterior temporal than in the occipital cluster (*p* = .012). The other factors and interactions did not reach significance (*p*>.05). Similarly, the N2 latency analysis revealed no significant main effects or interactions (>.05). ANOVA of N250 amplitudes showed a significant main effect of object category (*F*_(1,5)_ = 7.917, *p* = .037), corresponding to face-related stimuli eliciting smaller peaks than chair-related stimuli. Importantly, there was no significant difference in amplitude between the intact categorical stimuli and associated meaningless control stimuli (*F*_(1,5)_ = 1.774, *p* = .240). Regional cluster and the interaction terms did not reach significance (*p*>.05). The N250 latency analysis showed a significant main effect of regional cluster (*F*_(2,10)_ = 8.017, *p* = .008). Pairwise comparisons indicated that the N250 peaked significantly earlier in the parietal than in the posterior temporal cluster (*p* = .039), suggesting that dorsal stream processing precedes ventral stream activation during the perception of short-lived SFM stimuli. None of the other main effects or interactions were significant (*p*>.05).

**Figure 3:**
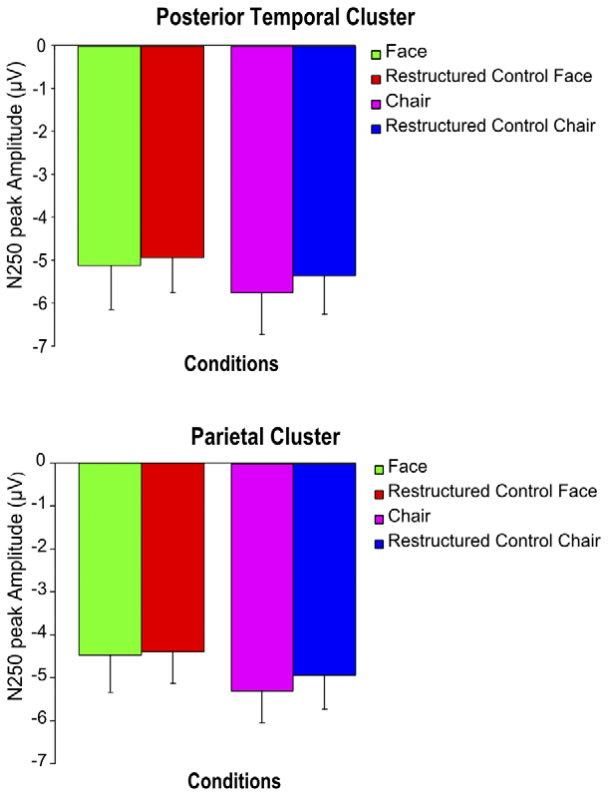
Visual features contribute to the amplitude of SFM-evoked ERP signals (Exp. 1A&B). Bar graphs depicting the mean N250 amplitudes for the SFM object and restructured object conditions of 14 participants pooled for the two durations used in experiment 1 and 2 (160 ms and 860 ms). The results demonstrate that there is no significant difference in the N250 amplitude between meaningful categorical objects and corresponding meaningless control objects in the posterior temporal and the parietal clusters. Error bars indicate the SEM.

To rule out that the absence of a difference between meaningful and matched meaningless control objects was due to an insufficient number of participants included in the analysis for the two stimulus durations separately, N250 peak amplitude values from all 14 participants who participated in experiments 1A and 1B were entered in a joint ANOVA. Within-subject factors were category, stimulus type, and cluster as well as the between-subject factor stimulus duration. Results revealed a significant main effect of category (*F*_(1,13)_ = 5.171, *p* = .041), no significant main effect of cluster (*F*_(2,26)_ = 2.698, *p* = .086) and most importantly no significant main effect of stimulus type (*F*_(1,13)_ = 2.913, *p* = .112), hence confirming results from the separate analyses of experiments 1A and B (Figure 3). It could be argued that increasing the number of participants beyond 14 would have revealed a significant effect. If true, our data suggest this effect would be small in size, at best. The between-subject factor duration and the interactions did not reach significance (*p*>.05).

In sum, the data show that by increasing the contribution of bottom-up processing more consistent differences in ERP amplitude between stimuli related to the chair-category and stimuli related to the face-category can be found at the level of the N250 component. However, we confirmed that the N2 and the N250 do not show an amplitude difference between intact SFM object stimuli and their restructured counterparts showing matched, meaningless objects. The data therefore suggest the possibility that lower-level visual features have a stronger influence on the amplitude of these ERP signals than the categorical aspect of object stimuli.

### Experiment 2A: Parametric variation of visual features in categorical stimuli: Psychophysics

**Figure 4:**
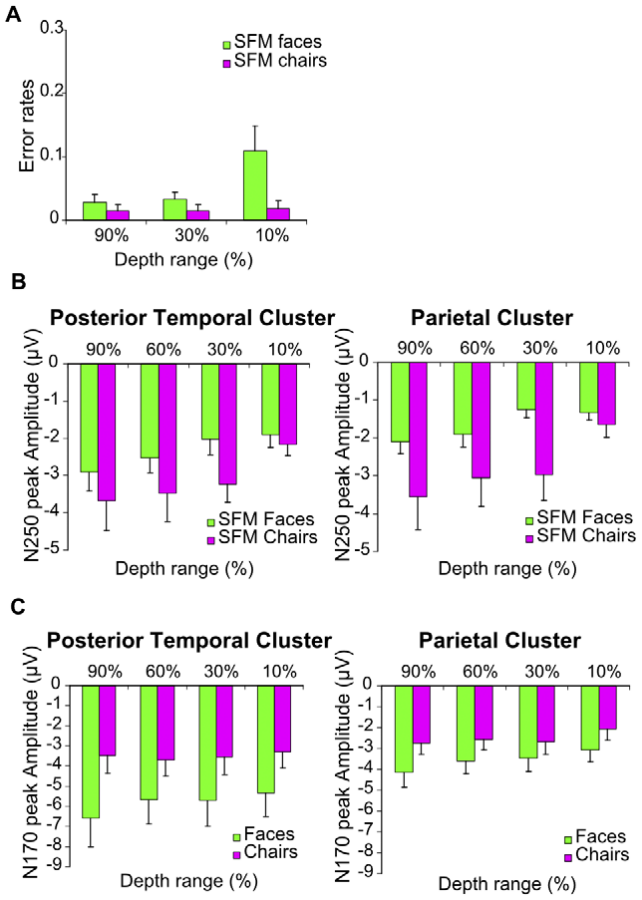
Depth range modulation of ERP signals (Exp. 2). **A** Behavioral data indicate that a reduction in depth range of the SFM stimuli decreased recognition performance of faces but not of chairs. Bar graphs indicate the error rate and error bars indicate the SEM. **B** The N250 is modulated by SFM face and chair depth range in both posterior temporal and parietal clusters. **C** The N170 to static faces but not to static chairs is modulated by depth range in posterior temporal and parietal cluster. N170 amplitude in the posterior temporal cluster is larger in response to faces compared to chairs. Bar graphs show the mean amplitude of the peaks and error bars the SEM.

In this experiment, we assessed the impact of visual feature manipulations induced by a reduction of depth range in SFM stimuli (160 ms) on behavioral task performance (Figure 4A). Overall error rates were low (<5%) indicating that participants were able to distinguish well between SFM stimulus categories (faces versus chairs). A repeated measures ANOVA testing the effect of the factors depth range (10%, 30%, 90%) and category (face, chair) showed a significant main effect of depth range (*F*_(2,40)_ = 10.354, *p*<.001) with post-hoc comparisons indicating that lowest depth stimuli resulted in higher error rates than highest depth stimuli (*p* = .001). In addition, there was a significant main effect of category (*F*_(1,20)_ = 19.799, *p*<.001) showing that participants committed less errors for the chairs than for the faces. Finally, we found a significant interaction between depth range and category (*F*_(2,40)_ = 10.379, *p* = .001). Post-hoc comparisons confirmed a strong effect of depth range for faces (*p*<.001) but no such effect for chairs (*p* = .956). Thus, these psychophysical data show that while depth range manipulations of SFM stimuli affect performance for face stimuli, they had no effect on chair stimuli.

### Experiment 2B: Parametric variation of visual features in SFM categorical stimuli: Effects on ERP amplitude

Experiments 1A and B have shown that the ERP components elicited by SFM-defined categorical stimuli (faces and chairs) were primarily driven by complex visual features that were preserved in the control stimuli, but in themselves did not produce meaningful categorical object perception. To more explicitly reveal the contribution of visual features to these ERP responses, we tested the effect of a parametric variation of depth range in SFM object stimuli (160 ms) on the amplitude of their ERP responses. This parametric variation was achieved by decreasing 3D stimulus depth (90%, 60%, 30%, 10%), thus reducing the relative contribution of complex 3D surface curvature cues and increasing the contribution of simpler 2D cues to object perception. The bar graphs in Figure 4B show the N250 peak amplitudes obtained in the different experimental conditions. A repeated measures ANOVA was performed with the factors depth range (4 levels), category (face, chair) and regional cluster (occipital, parietal, posterior temporal). Since the N2 was small or difficult to detect in some of the conditions, analysis was restricted to the early P100 component and the N250 waveform which was the larger, more consistent ERP peak and thus became the focus of our interest.

Results for the P100 amplitude showed no significant main effect of depth range, category or regional cluster (*p*>.05) and no significant interaction effects (*p*>.05). For the N250, the more depth was preserved in the stimulus, the larger the negativity was of the peak (see Figure 4B). This was confirmed by the significant main effect of depth range for the N250 peak (*F*_(3,18)_ = 5.015, *p* = .011) while neither category and cluster nor the interaction terms revealed significant main effects (all p's>.05). The N250 peak was hence significantly modulated by depth for both faces and chairs, whereas the behavioural data indicate an effect of depth range on categorization performance only for faces. Thus, the N250 amplitude varied with the depth/complexity of object-related visual features, in a way that was not directly related to categorical object perception or categorization performance.

### Experiment 2C: Parametric variation of visual features in luminance-defined categorical stimuli: Effects on ERP amplitude

The previous experiments indicated that variation in the SFM-related N250 ERP peak is determined to a great extent by complex visual feature information that is related to objects, but not by categorical object perception as such. Similar findings have also been obtained by showing sensitivity of the N170 to certain stimulus properties such as spatial frequency [17], [37] hence contradicting the claim of a direct specific link between the amplitude of ERPs and perception of object categories per se. However, here the psychophysical results suggest that the SFM depth range modulation influenced face categorization (error rates being higher for smaller depth ranges). The possibility hence remains that for the SFM face stimuli, the observed modulation of the N250 peak was at least in part due to the parametric destruction of categorical object perception instead of being attributable to reductions in the complexity and strength of visual object-related features. To test this possibility, we replicated the feature manipulation using luminance-defined chairs and faces (Figure 1B) (500 ms, details in Methods) for which the depth modulation reduced the complexity of visual features but did not have an effect on object recognition (*p*>.05) (error rates <1%, results not shown).

The ERP peaks of interest in this case were the P100 and the N170 (Figure 4C). Our repeated measures ANOVA for the P100 amplitude revealed no significant main effects of category or depth range (*p*>.05) but a significant main effect of cluster as well as an interaction between cluster and category (*F*_(2,8)_ = 4.824, *p* = .042 and *F*_(2,8)_ = 4.504, *p* = .024, respectively). Separate ANOVAs per category revealed a significant main effect of cluster for the faces (*F*_(2,8)_ = 6.086, *p* = .025) with the peak being larger in the occipital than in the posterior temporal cluster, but no such effect for the chairs (*F*_(2,8)_ = 3.398, *p* = .085). Analysis of the N170 peak amplitude revealed significant main effects of depth range (*F*_(3,12)_ = 4.018, *p* = .034) and cluster (*F*_(2,8)_ = 16.189, *p* = .002) while the factor category missed statistical significance (*p*>.05). Additionally, there was a significant interaction between category and cluster (*F*_(2,8)_ = 7.762, *p* = .034). A separate analysis only for face stimuli revealed a significant main effect of depth range (*F*_(3,12)_ = 4.217, *p* = .030), showing larger peak amplitudes for larger depth ranges (Figure 4C) as well as a significant effect of regional cluster (F_(2,8)_ = 14.419, *p* = .002), corresponding to larger peak amplitudes in the posterior temporal than in the parietal cluster. The interaction between depth range and cluster was not significant (*p*>.05). The same analysis at the N170 for chairs only revealed a significant effect of regional cluster (*F*_(2,8)_ = 13.637, *p* = .003) but no significant effect of depth (*F*_(3,12)_ = .224, *p* = .878) or interaction effect (*p*>.05).

In sum, we find that a reduction of stimulus depth range modulates the N170 response to faces without compromising object categorization as measured psychophysically. Additionally and in agreement with the current literature our results also indicated higher amplitudes of the N170 to static face compared to static chair stimuli in the posterior temporal cluster.

### Source localization

Source localization was performed for the SFM face and chair conditions in experiment 1B (with short duration SFM stimuli), separately for 5 of the 6 participants in this experiment. In the early time window (140–200 ms), we found in both conditions activity in dorsal regions, in the proximity of putative motion areas MT and V3 as well as activation in left superior parietal and right fusiform areas. In the later time window (200–290 ms) in addition to dorsal sources, the SFM object stimuli activated the right fusiform gyrus as well as regions around the right STS and the right lateral occipital cortex, consistent with dorsal-ventral integration in recognition of motion-defined objects [24], [38]. In all participants and for both conditions we observed a right hemispheric dominance. In sum, source localization of SFM stimuli yielded results suggesting a shift from sources for visual feature analysis in early ERPs, to more high-level sources in later ERPs.

## Discussion

In the present study, we investigated the perceptual correlates of ERP signals elicited by meaningful SFM- and luminance-defined objects. Since in SFM perception local motion cues define object categories, we asked whether category-specific responses could be isolated from visual feature-related response representations in the ERP signal. Interestingly, the SFM defined face and chair stimuli induced N2 and N250 peaks that showed little specificity for the categorical aspects of the stimuli. Instead, ERP peak amplitudes were highly sensitive to visual feature properties and were strongly modulated by visual stimulus feature manipulations.

These findings raise several questions: First, given the high impact of visual features on object-related ERP peaks in the present experiment, what general conclusions can we draw if an ERP peak varies its amplitude with object category? Second, can we interpret the N250 in reponse to SFM objects as a delayed N170? Third, how can we solve the inverse problem of relating an event-related potential with a stimulus that is characterized by a large set of dimensions on a variety of scales of complexity?

### The relative contribution of visual features and categorical aspects to object-related ERPs

Numerous studies have related ERP responses to category- (face-) specific processing (e.g. [3], [21], [39]). For this hypothesis to be supported, changes in the stimulus that change categorical perception should strongly modulate ERP amplitude, and changes in visual features that do not affect categorical perception should not or less strongly do so. However, in our data, we found the opposite, thereby raising the general question about contributions of low- to intermediate-level visual features in determining object-related ERPs. Our study does not deny the relative relevance of categorical processing but emphasizes the contribution of intermediate level features to ERP responses. The data we obtained pertain both to the N170 traditionally linked with categorical perception in static stimuli, and to the N250 peak we have observed for SFM objects.

Prior studies using static face stimuli have provided important insights into the extent to which ERPs can be linked to categorical perception by showing that removing outer face contours [40], presenting features in isolation [3] or changing the type of face representation (schematic, photographic etc) [23] resulted in N170 signal modulations, whereas changing the configuration of inner face components (ICs) without changing the contour of the face did not (e.g. [40]). These results indicate that changes of visual stimulus features result in N170 peak modulations while manipulations leaving these features and surface structures intact fail to do so. Moreover, the N170 to static faces shows less pronounced peak amplitudes for Mooney faces (made of simple object cues) compared to photographic and schematic faces [23] hence supporting this claim and indicating a role of visual feature complexity in determining ERP responses. This view is in line with results from our parametric feature manipulation experiment with static stimuli in which a decrease in depth range in face stimuli led to a significant decrease in N170 amplitude. Our findings indicate that a simple manipulation of visual depth cues in the image can modify N170 amplitudes in response to faces without affecting categorical perception, making an unambiguous interpretation of this ERP difficult. These findings complement similar results in previous studies pointing to a dependence of N170 category effects on parameters such as spatial frequency [37] and inter stimulus perceptual variance [41]. It should be acknowledged that our findings on the N170 are based on a relatively small pool of participants. Nevertheless, these results contribute to converging evidence from multiple experiments suggesting that a link between the amplitude of object related ERPs and categorical object perception is not straightforward.

Additionally, in the present study we observed an N250 component in response to SFM objects and investigated also for this component whether it can be linked unambiguously with categorical object perception. Here, we found again that a manipulation of depth range, causing a decrease in strength and complexity of several visual features but retaining the ‘categorical objectness’ of the stimuli led to a strong decrease in amplitude for the SFM-related N250. This effect was unlikely to be explained by changes in categorical perception as shown by psychophysical control data. In addition, the N250 ERP amplitudes were not significantly modulated by stimulus manipulations that destroyed categorical perception while keeping other lower level visual features constant.

The converging evidence suggests that from late ERP amplitudes (N170, N250) it is difficult to derive object-specificity since it depends on the values of visual parameters that within certain ranges do not influence object categorization but do modulate ERP amplitude. Other indicators such as the face-inversion effect [7], [8] of the N170 latency might therefore be a more reliable correlate of face-specific processing since in this manipulation most low-level visual properties remain intact while our face-processing expertise is destroyed. Nevertheless, since the ERPs of inverted stimuli also are likely to be affected by visual feature information, a parametric visual feature manipulation in inverted stimuli from different object categories might be useful to determine the robustness of the inversion effect as an indicator of face-specific processes.

### The N250: A delayed N170?

Among the numerous ERP studies that have aimed to investigate the time course of category-related effects in visual perception, to our knowledge, none have used meaningful and complex structure-from-motion 3D objects. With static stimuli, face-specific effects have mostly been reported at 170 ms [5], [6], [8] while in the present study using SFM-defined stimuli, no such face specificity was found in this time window. Our psychophysical experiments indicated that participants needed stimulus durations of at least 100 ms to identify object categories emerging from the moving dot pattern. This result is in line with a study comparing ERPs and behavioral responses to simple objects (sphere and cylinder) defined by luminance or motion, indicating a delay of 80 msec both in MEG/EEG responses and in reaction times when additional motion information was processed [42]. This suggests that the lack of a face-specific effect at 170 ms with SFM stimuli in the present study may be due to higher processing load which caused a shift of the component to 250 ms (N250). To test whether the N250 is in fact an N170 analogue, future studies could investigate face-inversion effects for SFM faces at this peak. Additionally, studies aiming to more accurately study the timing of SFM-related components could lock the ERP signals to button presses signaling categorization instead of stimulus onset. However, a latency shift of the N170 remains a matter of speculation and is not straightforward since the N250 was not face-specific. This lack of face specificity, however, might be due to a dominant contribution of lower-level and dynamic visual features to the ERP amplitudes, masking a relatively weak contribution of category-specific information, which in the context of our study would support the interpretation of the N250 as a delayed N170.

Irrespective of the precise interpretation of the N250, our study indicates that N250 amplitudes in response to SFM objects cannot easily be linked with object categorization, given their strong dependency on visual cues in the image that however do not affect categorization performance.

### Inverse problem

Categorical stimuli constitute a challenge for the analysis of their ERP responses since they are characterized by a large set of stimulus dimensions. Object category, cue configurations, cue complexity/depth, dynamic features as well as high-level factors may each contribute to the observed signal posing an interesting inverse problem. Below, we briefly discuss contributions from a subset of relevant factors.

The N2 we report here has been described in previous studies investigating the ERP responses to motion onset perception [43]–[45] and is believed to originate from MT [46]. The human motion complex hMT+ [47], [48] in its medial superior temporal location has repeatedly been shown to play a role in SFM perception [49]–[51]. However, also at later latencies motion-related effects have been reported. An additional negative peak at 240 ms over occipitotemporal regions which, in contrast to the N2 is believed to reflect the higher processing of motion stimuli [52], may also be contributing to the N250 peak amplitude in the present study.

Effects of depth cues on the ERP signal have previously been reported for static stimuli indicating a negative potential around 170 ms present on occipital sites which was consistently enhanced to depth cues [53]. Furthermore, fMRI revealed that area hMT+ is part of a network with right hemispheric dominance including a lateral occipital region, five sites along the intraparietal sulcus (IPS), and two ventral occipital regions that is involved in extracting depth from motion [49]. The depth modulations of the ERP peaks in the present study are likely to be driven by computations taking place in this entire network.

Although there seems to be a strong influence of feature-based properties on ERP signals, it is not the case that ERPs cannot pick up category-related effects. Paradigms using stimuli in which category percepts pop out once certain structures/configurations are detected without any change in the stimulus' physical features, provide strong evidence for more isolated contributions of categorical object classification to ERP signals [54]–[56].

Hence, many visual features in an object stimulus could influence the amplitude of ERPs in addition to its categorical aspect. Although the data we have obtained were collected in a set of experiments with relatively small numbers of participants in the individual experiments, the converging results from the data as a whole (on 27 participants) support this idea. They indicate that the amplitude of late ERP components that are elicited by object stimuli is difficult to link unambiguously with categorical object perception. This is shown both by the lack of ERP amplitude modulation when specific categorical perception is destroyed but low-to-mid-level visual features are retained and by the strong and significant ERP amplitude modulations in stimuli that retain their categorical aspect but that are changed on the strength of low-to-mid-level visual features. Thus, our data indicate that the contributions of categorical and visual processing are difficult to distinguish, pointing to a severe inverse problem in interpreting those ERP components. While measuring changes in categorical perception in physically unchanged stimuli may be one approach to tackle this problem, new ways of analyzing ERP data that no longer rely on trial averaging may provide another. Specifically, Bayesian approaches and classification algorithms like SVMs [57]–[59] might be promising tools to overcome the inverse problem faced by many studies in the ERP field.
